# Hematologic manifestations in a child with HIV; a Case Report

**Published:** 2012-09-22

**Authors:** Sh Osiya, F Binesh, F Ferdosian, M Shakiba

**Affiliations:** 1 Department of pediatrics and pathology, Shahid Sadoughi University of Medical Science and Health Services, Yazd, Iran.

**Keywords:** HIV, Child, Signs and Symptoms

## Abstract

**Background:**

Immune deficiency in human might be primary or secondary and could be seen with a wide variety of manifestations. In the following, we presented a Child with various complains that diagnosed to have HIV infection.

**Case Report:**

A 2/5 y/o child was admitted to the hospital for FUO with prolonged cough, FTT, cervical lymphadenopathy, hepatosplenomegaly and bilateral optic neuritis. . He was hospitalized for fever, cytopenia and hepatosplenomegaly one year ago, and three months later in an outpatient visit, these signs improved, except thrombocytopenia. In evaluation, bicytopenia, elevated ESR, hyperlipidemia, hyperproteinemia, thrombosis of the transverse sinus of brain, antiphospholipid antibodies , decreased levels of protein S and factor V Leiden and increased level of anti thrombin III were detected. Consequently, the result of HIV antibody showed positive. In addition to warfarin and cotrimoxazole therapy, he was referred to special center for possible HARRT therapy.

**Conclusion:**

In approach to patients with various clinical presentations such as cytopenia, recurrent or persistent lymphadenopathy, unexplained hyperproteinemia or hyperlipidemia, evaluation of HIV infection is highly recommended for consideration and further therapy.

## Introduction

Immune deficiency in human might be primary or secondary to malnutrition, radiotherapy, Immunosuppressive agents, infections and metabolic diseases. Many disorders such as dermatologic, cardiac , renal, hematologic, gastrointestinal (1,2) and neurologic problems in addition to common and/or opportunistic infections have been seen in these patients, so in approach to lots of medical problems, evaluation for immune deficiency should be kept in mind. In the following, we present a child with secondary immune deficiency who came to us for multiple clinical complains. 

## Case presentation

A 1/5 year old boy was hospitalized for lymphadenopathy and low grade fever for 1 week. His weight was 7 kg. In physical examination, multiple lymphadenopathy in cervical and inguinal areas together with hepatosplenomegaly were diagnosed. He was the second child of non relative parents. The first child of family died because of respiratory infection at the age of 6 months old. Results of Para clinic evaluation were reported as below:

WBC /9000, Hb/8/5. Plt /51000, ESR/92, CRP/Neg, LDH/normal , TG/277, uric acid /3, AST/36, ALT/14, Alp/306, Total bilirubin /0.4, Total protein/8.7 Alb/4.6, Urea/ 27, Cr/0.5, Urine analysis. Stool examination, Bone marrow aspiration and CXR were all normal.

Abdominal sonography / enlarged liver and spleen with normal echo, liver biopsy/Mononuclear cell infiltration in portal tracts, in addition to expanded fibrotic spaces and mild to moderate piecemeal necrosis. Immunologic and virology assessment were requested, but his parents refused to do it, and as a result he was discharged. In an outpatient visit, in 3 months later, he improved significantly, but thrombocytopenia had been persisted. They did not return for follow up till nine months later. One year after the first visit, the boy was hospitalized again with FUO and protracted cough for 20 days. In physical examination weight was 9 kg, Height/80 cm and head circumference/ 47cm; all of them were below 3 percentile. Hepatosplenomegaly and cervical lymphadenopathy were apparent. We thought about viral infections, TB, salmonellosis, malignancy, immune deficiency and collagen vascular diseases for this patient. Para clinic data have shown as following: WBC/8000, Hb/8.4, plt/147000, ESR/97, CRP/negative, TG/353, Total protein /9.8.AST/31, ALT/26, ALP/286.Alb/3.2, Bil/0.3, LDH/838, Retic/2%, Coombs/ negative and EBV( Ig M and IgG) was found to be Positive. Abdominal sonography showed enlargement of liver and spleen but bone marrow aspiration was normal with no acid fast bacillus , PPD test / negative and lymph node biopsy showed reactive lymphadenitis , flow cytometry / CD4: 26.8,CD3: 75,CD16: 10.2,CD19: 4.5,CD56 : 9 ,CD18: 95. In ophthalmologic consultation, bilateral optic neuritis was seen, and brain MRI showed thrombosis of right transverse sinus. Echocardiography was normal, but antipospholipid antibodies were present and decreased level of protein S and factor 5 Leiden and increased level of anti thrombin III was detected. HIV antibody was reported positive in the patient and his parents. By reviewing the family history, it was apparent that the patient s father had been drug abuser from two years before. Therapy with Warfarin and Cotrimoxazole was prescribed for the patient and they were referred to the special center for further treatment.

## Discussion

 HIV invades to the immune system, and gradually destroys CD4 lymphocytes. As a result immune system becomes suppressed and the patient become susceptible to various infections (3) .Our case had prolonged fever and active EBV infection. 

In children, the immaturity of immune system accelerates disease progression, decrease the long of each clinical stage and, if not treated properly, 50% of them die before the second year of life (3). 

Lymphadenopathy, often with hepatosplenomegaly might be the first presentation of HIV infection. During the first year of life, oral candidiasis, FTT and diarrhea are the common signs usually observed in most patients (5). In our patient, FTT, lymphadenopathy and hepatosplenomegaly were the earliest symptoms. HIV infection causes hyperproteinemia in 60% of non elderly patients, so we should rule out HIV in patients with hyperproteinemia (4). Our case had total protein 8.7 in the first period of hospitalization and 9.8 in the second time.

Hematologic indices in Aids patients represent that 60% of them suffered from anemia and lymphopenia, 50% neutropenia and 40% thrombocytopenia (6). Incidence of cytopenia is directly related to the severity of immune suppression. HIV infection should be ruled out in every patient with any type of cytopenia (7). Our child had anemia and thrombocytopenia in both episodes of hospitalization. Coagulation abnormalities such as thrombosis (8), anti phospholipids antibodies, and decreased level of protein C and S (9, 10 and 11) might be seen in HIV infection; as all were detected in this patient. Because the GI tract is a common route for HIV entrance, gastrointestinal lymphoid tissue is severely damaged in HIV infection (16). HIV enteropathy presents with chronic diarrhea, malnutrition and weight less (12). Opportunistic infections and malignancies involve the GI tract and liver too. With progression of the disease, the virus disseminates in hepatic kupffer cells (13). Liver histology shows nonspecific portal inflammation, kupffer cell hyperplasia and high incidence of opportunistic infections (14, 15). In our case, FTT and hepatosplenomegaly were seen as significant findings and liver biopsy showed mononuclear cell infiltration in portal tracts, in addition to expanded fibrotic spaces and mild to moderate piecemeal necrosis. CNS problems may be occurred in more than 40% of HIV infected persons and in 10-20% of cases are the first presentations. In autopsy, incidence of neurologic disorders has been reported to be 80% (17). Incidence of encephalopathy in HIV infected children is about 13-23 % (18). Other uncommon but important neurologic disorders are vasculitis, aneurisms, atherosclerosis and vascular occlusion (19). In this report, thrombosis of transverse sinus and bilateral optic neuritis existed. So in our patient, hematologic presentation (cytopenia and recurrent lymphadenopathy), coagulation abnormalities (decreased protein S and factor V leiden, increased antithrombin 3 in addition to anti phospholipid antibodies), neurologic symptoms (thrombosis of transverse sinus and bilateral optic neuritis), gastrointestinal symptoms (FTT and recurrent hepatosplenomegaly), prolonged fever and active EBV infection were detected.

## Conclusion

In approach to patients with various clinical presentations such as cytopenia, recurrent or persistent lymphadenopathy, Recurrent or chronic hepatosplenomegaly, FUO, vascular problems in CNS, unexplained hyperproteinemia or hyperlipidemia, evaluation of HIV infection is highly recommended for consideration.
